# Cytotoxic Effects of Milled, 3D-Printed, and Conventional Methacrylate-Based Resins After Exposure to Denture Cleansers: A Preliminary In Vitro Screening Study

**DOI:** 10.3390/ma19214761

**Published:** 2026-11-09

**Authors:** Rumeysa Bayram Büyükokuroğlu, Gülsüm Sayın Özel

**Affiliations:** Department of Prosthodontics, School of Dentistry, Istanbul Medipol University, Istanbul 34810, Türkiye; gozel@medipol.edu.tr

**Keywords:** denture base resin, denture cleansers, PMMA, CAD/CAM, 3D printing, cytotoxicity, human gingival fibroblasts

## Abstract

This study evaluated cellular responses to extracts of CAD/CAM-milled PMMA, 3D-printed methacrylate-based resin, and conventionally heat-polymerized PMMA after denture cleanser exposure. Specimens (MTT, *n* = 240; BrdU, *n* = 36) were assigned to Corega, chlorhexidine, Listerine, or distilled water. Following accelerated cumulative exposure (1825 min; not simulating clinical use), extracts were applied to human gingival fibroblasts. Cellular metabolic activity was assessed by MTT absorbance, DNA synthesis-associated proliferative activity by quantitative BrdU immunofluorescence, and morphology and F-actin organization qualitatively. Material and cleansing solution significantly affected MTT absorbance (both *p* < 0.001), with a significant interaction (*p* = 0.008). CAD/CAM-milled and 3D-printed materials exhibited higher MTT absorbance than conventional PMMA (*p* < 0.001 and *p* = 0.003, respectively), without a significant difference between them (*p* = 0.071). Distilled water yielded higher MTT absorbance than all cleansers (all *p* < 0.001). BrdU labeling showed a significant cleansing solution effect (*p* < 0.001) but no significant material effect or interaction. In this preliminary screening study, cleansing solution affected both metabolic and DNA synthesis-associated responses, whereas material differences were significant only for MTT absorbance. Mechanisms were not investigated; the milled material (TEMPO-CAD) is not primarily a denture base material, so findings should not be generalized.

## 1. Introduction

Polymethyl methacrylate (PMMA) is widely used as a denture base material because of its ease of processing, adequate mechanical properties, and favorable esthetic characteristics [1,2].

Recent developments in digital dentistry have introduced alternative denture base fabrication techniques, including CAD/CAM milling and three-dimensional (3D) printing [3]. CAD/CAM fabrication has been shown to improve denture base adaptation, with CAD/CAM-fabricated dentures demonstrating greater congruence with denture-bearing tissues than conventionally fabricated dentures [4]. Additive manufacturing techniques have also been investigated for the fabrication of dental prostheses [5]. Maintaining denture hygiene is essential for limiting biofilm accumulation and denture-related complications, and chemical denture cleansers are commonly recommended as part of denture care [6]. Previous studies have investigated the effects of denture cleansers on conventional and digitally fabricated denture base materials, including their physical, mechanical, and biological properties [7,8,9,10]. The present study extends this work by comparatively evaluating the cellular responses to conventionally polymerized, CAD/CAM-milled PMMA, and 3D-printed denture base resin extracts after exposure to different cleansing solutions under the same experimental conditions. Corega is a commercial effervescent denture cleanser, whereas chlorhexidine and Listerine are not formulated as denture cleansers (chlorhexidine is an antiseptic and Listerine is an essential-oil mouthrinse); they were included as antimicrobial agents that are also used by some patients and clinicians for denture hygiene [11,12]. In vitro cell-based assays are commonly used to evaluate the biological response to dental materials [13,14]. The MTT assay provides quantitative information on cellular metabolic activity, while immunofluorescence imaging can provide complementary information on cellular morphology and proliferation-related staining. BrdU staining can be used to identify cells undergoing DNA synthesis [15].

Therefore, this study aimed to compare the in vitro response of human gingival fibroblasts to extracts from a CAD/CAM-milled prepolymerized PMMA material, a 3D-printed methacrylate-based denture-base resin, and a conventionally heat-polymerized denture-base PMMA after accelerated cumulative exposure to different denture cleansing solutions. Cellular metabolic activity was assessed using the MTT assay, while BrdU immunofluorescence was used to quantitatively assess DNA synthesis-associated proliferative activity. Cellular morphology and F-actin organization were additionally evaluated qualitatively. This study should be regarded as a preliminary screening study: it was designed to detect differences in cellular responses among materials and cleansing conditions, not to explain them, and the mechanisms underlying any observed differences are not yet known and were not investigated.

## 2. Materials and Methods

**Figure 1:**
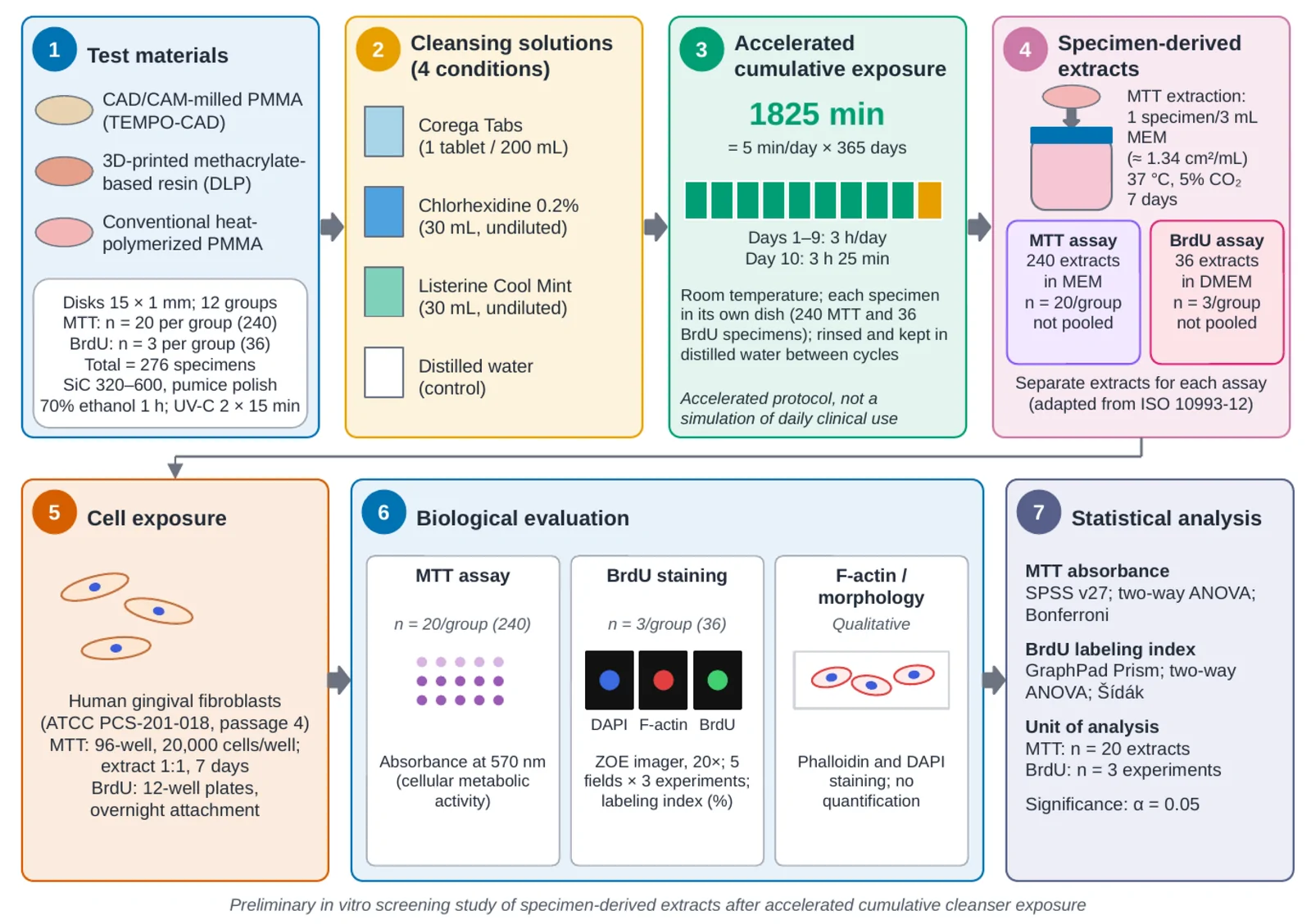
Schematic illustration of the experimental design and workflow. Three resin materials were subjected to four cleansing conditions, resulting in 12 experimental groups. A total of 276 specimens were used: 240 specimens for the MTT assay (*n* = 20 per group) and 36 additional specimens for BrdU immunofluorescence analysis (*n* = 3 per group). Specimens underwent an accelerated cumulative cleansing protocol totaling 1825 min, rather than a simulation of daily clinical use. For the MTT assay, 240 independent specimen-derived extracts were prepared using Minimum Essential Medium (MEM), whereas 36 additional extracts were prepared independently using Dulbecco’s Modified Eagle Medium (DMEM) for BrdU analysis.The extraction procedure was adapted from ISO 10993-12 [16]. All extracts were maintained separately without pooling. Human gingival fibroblasts were exposed to the specimen-derived extracts. MTT absorbance was measured to assess cellular metabolic activity, whereas BrdU-positive nuclei were quantified to evaluate DNA synthesis-associated proliferative activity. Cellular morphology and F-actin organization were assessed qualitatively. MTT absorbance and BrdU labeling indices were analyzed separately using two-way ANOVA, with Bonferroni-adjusted and Šídák-adjusted comparisons, respectively. Abbreviations: ANOVA, analysis of variance; BrdU, 5-bromo-2′-deoxyuridine; CAD/CAM, computer-aided design/computer-aided manufacturing; DAPI, 4′,6-diamidino-2-phenylindole; DLP, digital light processing; DMEM, Dulbecco’s Modified Eagle Medium; MEM, Minimum Essential Medium; MTT, 3-(4,5-dimethylthiazol-2-yl)-2,5-diphenyltetrazolium bromide; PMMA, polymethyl methacrylate; SiC, silicon carbide; UV-C, ultraviolet C. Icons are schematic.

A schematic overview of the experimental design and workflow, including specimen fabrication, accelerated cumulative cleansing exposure, separate extract preparation for MTT and BrdU analyses, cell culture, biological evaluation, and statistical analysis, is presented in Figure 1.

### 2.1. Study Design and Sample Size

An a priori power analysis was performed using G*Power software (version 3.1.9.2) [17]. Based on a significance level of α = 0.05, a statistical power of 80% (1 − β = 0.80), and a medium effect size (f = 0.25) according to Cohen [18], the minimum required sample size for the planned two-way ANOVA was calculated to be 225 specimens.

### 2.2. Specimen Preparation

Disk-shaped specimens (15 mm in diameter and 1 mm in thickness) were designed using Blender software (v3.6, Blender Foundation, Amsterdam, The Netherlands). The peripheral edges of the specimens were rounded to standardize specimen geometry. The specimens were divided into three groups according to the manufacturing method: CAD/CAM-milled PMMA, 3D-printed methacrylate-based denture base resin, and conventionally heat-polymerized PMMA. For the two digitally fabricated groups, the designs were exported in standard tessellation language (STL) format for subsequent manufacturing.

1. Additive Manufacturing (3D Printing) Group: The 3D-printed specimens were fabricated from a methacrylate-based photopolymer denture base resin (Cyclone Denture, Dark Pink, On Dent, Istanbul, Türkiye; Lot DPD-1905082) using a SolFlex 250 Ultra HD UV-LED DLP 3D printer (W2P Engineering GmbH, Vienna, Austria; supplied by Onex Dental, Istanbul, Türkiye) at a wavelength of 385 nm and a layer thickness of 50 μm. After printing, the specimens were pre-cleaned for 3 min in a 99% isopropyl alcohol (IPA) ultrasonic bath, followed by a second 3 min ultrasonic wash in fresh 99% IPA. The specimens were then air-dried and post-polymerized using an Otoflash G171 unit (NK-Optik, Baierbrunn, Germany), with 2000 flashes applied to each side (4000 flashes in total).
2. Subtractive Manufacturing (Milling) Group: Specimens were milled from prepolymerized polymethyl methacrylate (PMMA) blocks (TEMPO-CAD, Pink, Lot No. 21127, On Dent, Istanbul, Türkiye) using a 5-axis milling machine (CORiTEC 150i, imes-icore GmbH, Eiterfeld, Germany).
3. Conventional Manufacturing (Heat-Polymerized) Group: The conventionally heat-polymerized specimens were fabricated using a heat-polymerized PMMA denture base resin (LUNA ACRYL HEATCURE, Bedir Dental, Istanbul, Türkiye). Wax patterns (15 mm in diameter and 1 mm in thickness) were invested in metal flasks using Type III dental stone. Following wax elimination, the resin was mixed and packed into the molds during the dough stage. The water temperature was gradually increased to 100 °C, followed by polymerization at 100 °C for 30 min, in accordance with the manufacturer’s recommended polymerization protocol. After polymerization, the flasks were allowed to cool before deflasking.

After fabrication, all specimens were sequentially finished using 320-, 400-, and 600-grit silicon carbide abrasive papers and polished with pumice. The specimens were then immersed in 70% ethanol for 1 h and subsequently dried in a UV-equipped sterile cabinet. UV-C irradiation was applied for 15 min on each side under laminar-flow conditions (30 min total exposure). Disinfection with ethanol followed by UV irradiation has been used before cytotoxicity testing of denture base resins [19,20]. Because ethanol can affect the surface of denture base polymers in a time- and concentration-dependent manner [21], a possible influence on surface structure and on the leachable content of the specimens cannot be excluded; UV irradiation alone has been used in a comparable study [22]. The dimensions of the specimens were verified using a digital caliper to ensure standardization.

### 2.3. Denture Cleansing Protocol

The specimens were assigned to four cleansing conditions, resulting in 12 experimental groups according to the combination of denture base material and cleansing solution (*n* = 20 specimens per combination for the MTT assay and *n* = 3 additional specimens per combination for BrdU analysis; 276 specimens in total).

The denture cleansing agents, their manufacturers, compositions, and immersion protocols are summarized in Table 1.

To achieve a cumulative exposure duration corresponding to 5 min of daily cleansing for 365 days [11,23], a total exposure time of 1825 min was used in an accelerated cumulative exposure protocol. For experimental feasibility, this cumulative exposure was distributed over 10 consecutive days, with specimens immersed for 3 h per day for the first 9 days and for 3 h 25 min on day 10. This accelerated protocol was intended to reproduce the cumulative exposure duration associated with one year of daily cleansing rather than to replicate the temporal pattern of clinical use. Fresh cleansing solutions were prepared for each daily exposure cycle, and all immersion procedures were performed at room temperature. Each specimen was immersed individually in a separate transparent Petri dish containing the respective cleansing solution; thus, 276 Petri dishes were used in total (240 for the MTT specimens and 36 for the BrdU specimens), and all specimens were subjected to the same cleansing protocol. After each exposure cycle, the specimens were rinsed under running water and stored in distilled water at room temperature until the next exposure cycle. The control specimens were maintained in distilled water throughout the experimental period.

### 2.4. Human Gingival Fibroblast Culture

Human gingival fibroblasts (ATCC PCS-201-018; American Type Culture Collection, Manassas, VA, USA) were incubated at 37 °C in a humidified atmosphere containing 5% CO_2_, and fourth-passage (P4) cells were used for all experiments. Routine mycoplasma testing was performed on both the cell cultures and the culture media (MEM supplemented with fetal bovine serum [FBS]) using the EZ-PCR Mycoplasma Detection Kit (Sartorius, Göttingen, Germany). The tests were performed regularly to assess potential mycoplasma contamination. Independent experiments were conducted using cells obtained from separate culture runs.

The cytotoxic effects of extracts derived from various dental materials on human gingival fibroblast (HGF) cells were evaluated using the MTT [3-(4,5-dimethylthiazol-2-yl)-2,5-diphenyltetrazolium bromide] cell viability assay. All experimental procedures were performed under sterile conditions within a laminar flow cabinet.

Prior to specimen placement, sterile Petri dishes were disinfected with 70% ethanol and exposed to ultraviolet (UV) light in a laminar flow cabinet for 15 minutes. All subsequent procedures, including specimen transfer and cell culture preparation, were performed under aseptic conditions within the laminar flow cabinet.

### 2.5. Preparation of Material Extracts

The extraction procedure was adapted from the general principles of ISO 10993-12 [16]. Each specimen was individually placed in a separate well of a 6-well culture plate containing 3 mL of minimum essential medium (MEM), ensuring that the specimen was completely submerged. Based on the total geometric surface area of each disk (approximately 4.01 cm^2^), the surface-area-to-extraction-medium ratio was approximately 1.34 cm^2^/mL. This ratio was lower than the ratio commonly recommended in ISO 10993-12 for specimens of this thickness (approximately 3 cm^2^/mL) and used in comparable denture base studies [22]. The extracts were additionally diluted 1:1 before cell exposure (Section 2.6). The same extraction ratio was applied to all experimental groups, ensuring consistency in the extraction conditions; however, the resulting extracts were less concentrated than those prepared using the recommended ratio, which may have reduced the sensitivity of the assay.

Twenty independent specimen-derived extracts were obtained for each of the 12 experimental groups, resulting in a total of 240 individual extracts. All extracts were maintained separately throughout the experiment and were not pooled at any stage. The specimens were incubated in MEM at 37 °C in a humidified atmosphere containing 5% CO_2_ for 7 days. During incubation, the plates were partially sealed with Parafilm^®^ to minimize evaporation while maintaining gas exchange. After incubation, each extract was collected separately and stored at 4 °C until further analysis.

### 2.6. MTT Assay

For cytotoxicity evaluation, fourth-passage human gingival fibroblasts (HGF) were seeded in 96-well plates at a density of 20,000 cells per well. After cell attachment, the prepared material extracts were diluted 1:1 with culture medium and applied to the cells. Each specimen-derived extract was applied to a separate well, resulting in 20 MTT measurements per experimental group (*n* = 20). Extracts from different specimens were not combined before or during the MTT assay.

The cells were incubated with the extracts at 37 °C in a humidified atmosphere containing 5% CO_2_ for 7 days. At the end of the exposure period, MTT [3-(4,5-dimethylthiazol-2-yl)-2,5-diphenyltetrazolium bromide] reagent (Sigma Chemical Co., St. Louis, MO, USA) was added. Following incubation with the MTT reagent, the resulting formazan crystals were dissolved, and optical density was measured at 570 nm using a microplate reader (SPECTROstar Nano; BMG LABTECH, Ortenberg, Germany).

The numerical values used for the inferential statistical analyses were the measured MTT absorbance values, and MTT absorbance was used as an indicator of cellular metabolic activity. Cell viability percentages were calculated from the MTT absorbance values using the blank-corrected untreated cell control according to the following equation:

Cell viability (%) = [(A_sample_ − A_blank_)/(A_control_ − A_blank_)] × 100

where A_sample_ is the absorbance of cells exposed to the specimen-derived extract, A_blank_ is the absorbance of the cell-free blank, and A_control_ is the absorbance of untreated cells cultured in medium.

The same reference values were used for all experimental groups. The control absorbance (A_control)_ was calculated as the mean absorbance of 20 untreated control wells across plates, whereas the blank absorbance (A_blank_) was calculated as the mean absorbance of 20 cell-free blank wells. These common reference values were used to calculate the cell viability percentages for all 240 specimen-derived measurements.

Cell viability percentages were used as descriptive values for comparison with previously published cytotoxicity studies; the inferential statistical analyses of the present study remained based on the measured MTT absorbance values. All biological analyses were conducted at NeoPreklinik Laboratories, Biruni University Technopark, Istanbul, Türkiye.

### 2.7. BrdU Immunofluorescence Staining

DNA synthesis-associated proliferative activity was evaluated using 5-bromo-2′-deoxyuridine (BrdU) immunofluorescence staining according to the manufacturer’s protocol (Thermo Fisher Scientific, Waltham, MA, USA). For the BrdU experiments, 36 new material specimens were prepared, representing three independent specimens for each of the 12 material–cleansing solution combinations. Fresh specimen-derived extracts were prepared individually using Dulbecco’s Modified Eagle Medium (DMEM). These extracts were maintained separately without pooling and were prepared independently of the 240 specimen-derived extracts used for the MTT assay, which were obtained using Minimum Essential Medium (MEM).

A 10 mM BrdU stock solution was prepared in anhydrous dimethyl sulfoxide (DMSO) and diluted in prewarmed culture medium to obtain a final labeling concentration of 10 µM. Cells were incubated with 10 µM BrdU for 2 h at 37 °C and subsequently washed with phosphate-buffered saline (PBS).

Cells were fixed with 3.7% formaldehyde in PBS for 15 min at room temperature and permeabilized with 0.1% Triton X-100 for 20 min. To facilitate antibody detection of incorporated BrdU, DNA was denatured by sequential incubation with 1 N HCl on ice for 10 min and 2 N HCl at room temperature for 10 min, followed by neutralization with phosphate/citric acid buffer (pH 7.4) for 10 min. Cells were then incubated with a fluorophore-conjugated anti-BrdU antibody; no secondary antibody was used.

Two staining approaches were used. For quantification, BrdU was detected with a Texas Red-conjugated anti-BrdU antibody, and nuclei were counterstained with DAPI. BrdU-positive nuclei and total nuclei were scored directly under a ZOE Fluorescent Cell Imager (Bio-Rad Laboratories, Hercules, CA, USA) at 20× magnification in five randomly selected, non-overlapping fields per independent experiment. Three independent experiments were performed for each material–cleansing solution combination (*n* = 3), using cells obtained from separate culture runs. Scoring was performed directly at the microscope by an investigator blinded to the experimental group identities, and images were not acquired for every scored field. A nucleus was classified as BrdU-positive when distinct intranuclear staining could be distinguished from background fluorescence by comparison with the negative control. Counts from the five fields were combined to calculate one BrdU labeling index per independent experiment using the following equation:

BrdU labeling index (%) = (Number of BrdU-positive nuclei/Total number of nuclei) × 100.

The BrdU labeling indices from the three independent experiments were summarized as mean ± standard deviation (SD).

For qualitative illustration of nuclear BrdU incorporation and cytoskeletal organization, an additional double-staining experiment was performed in which BrdU (green) was combined with phalloidin staining of F-actin (red) and DAPI nuclear counterstaining (blue). Images were acquired at 20× with the same imager; representative regions were selected and enlarged, and the individual channels were merged to prepare the composite panels shown in the Results section. Identical brightness and contrast adjustments were applied uniformly to all 12 panels (A1–D3) of the composite figure, without selective enhancement of any panel or region. These images are provided for qualitative illustration only; quantification was based exclusively on the direct microscopic counts described above. Cellular morphology and F-actin organization were evaluated qualitatively.

### 2.8. Statistical Analysis

MTT absorbance data were analyzed using IBM SPSS Statistics for Windows, version 27.0 (IBM Corp., Armonk, NY, USA). Each specimen-derived extract was considered an independent experimental unit, yielding 20 observations per material–cleansing solution combination (*n* = 20). Descriptive statistics were expressed as mean ± standard deviation (SD), minimum, and maximum. MTT data were assessed using the Shapiro–Wilk and Levene tests, followed by two-way ANOVA for the effects of material, solution, and their interaction; Bonferroni adjustment was applied to pairwise comparisons. BrdU labeling indices were analyzed using GraphPad Prism (version 11.1.0.218; GraphPad Software, Boston, MA, USA). The independent experiment, rather than the microscopic field, was the unit of analysis (*n* = 3 per material–solution group). Values were expressed as mean ± SD and evaluated by two-way ANOVA, followed by Šídák-adjusted comparisons between cleansing solutions averaged across materials. BrdU ANOVA residuals were evaluated using the Shapiro–Wilk test. Variance heterogeneity was explored using the original mean-centered Levene test and the median-centered Brown–Forsythe variant across the 12 groups.

Statistical significance was set at *p* < 0.05.

## 3. Results

The descriptive statistics for MTT absorbance values according to denture base material and cleansing solution are presented in Table 2. Mean, standard deviation, minimum, and maximum values were calculated for each experimental group. Mean MTT absorbance values ranged from 0.72 ± 0.22 to 1.26 ± 0.31 across the experimental groups. The highest mean absorbance was observed for CAD/CAM milled specimens immersed in distilled water (1.26 ± 0.31), whereas the lowest was recorded for conventionally fabricated PMMA specimens immersed in Corega (0.72 ± 0.22). Raw MTT absorbance data are provided in Supplementary Table S2.

Two-way ANOVA demonstrated significant main effects of denture base material (F = 15.936, *p* < 0.001) and cleansing solution (F = 12.971, *p* < 0.001). A significant denture base material × cleansing solution interaction was also detected (F = 3.003, *p* = 0.008), indicating that the effect of cleansing solution on MTT absorbance differed according to denture base material (Table 3).

Bonferroni post hoc comparisons showed that CAD/CAM milled and 3D-printed specimens exhibited significantly higher MTT absorbance values than conventionally fabricated PMMA specimens (*p* < 0.001 and *p* = 0.003, respectively), whereas no significant difference was observed between CAD/CAM milled and 3D-printed specimens (*p* = 0.071). Distilled water resulted in significantly higher MTT absorbance values than Corega, chlorhexidine, and Listerine (all *p* < 0.001), whereas no significant differences were observed among the three cleansing solutions (all *p* > 0.05).

Because of the significant interaction, pairwise comparisons were also performed within each factor. Under Corega immersion, CAD/CAM milled specimens exhibited significantly higher MTT absorbance values than both 3D-printed (*p* = 0.024) and conventionally fabricated PMMA specimens (*p* < 0.001). Under distilled water immersion, both CAD/CAM milled and 3D-printed specimens demonstrated significantly higher values than conventionally fabricated PMMA specimens (both *p* < 0.001). Within the CAD/CAM milled group, Corega resulted in significantly higher MTT absorbance than Listerine (*p* = 0.045), whereas chlorhexidine and Listerine resulted in significantly lower values than distilled water (*p* = 0.001 and *p* < 0.001, respectively). Within the 3D-printed group, Corega, chlorhexidine, and Listerine resulted in significantly lower MTT absorbance values than distilled water (*p* < 0.001, *p* < 0.001, and *p* = 0.001, respectively). No significant differences among cleansing solutions were observed within the conventionally fabricated PMMA group (Table 4). Complete Bonferroni-adjusted pairwise comparisons, including nonsignificant comparisons, are provided in Supplementary Table S1.

**Figure 2:**
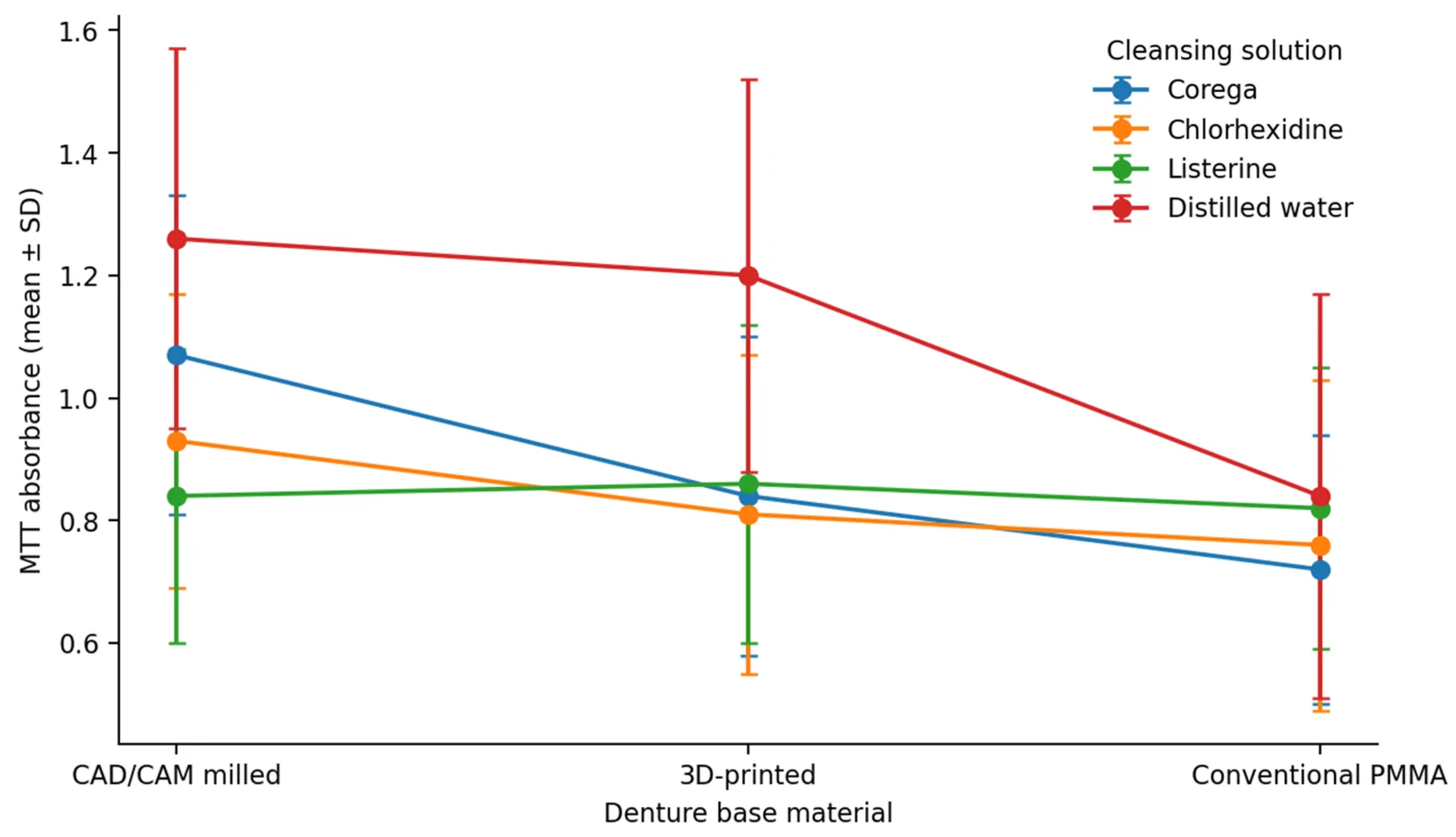
Interaction plot illustrating the effects of denture base material and cleansing solution on MTT absorbance values. Data are presented as mean ± standard deviation. A significant interaction between denture base material and cleansing solution was observed (*p* = 0.008).

As illustrated in Figure 2, the effect of cleansing solutions on MTT absorbance varied according to denture base material. Distilled water yielded the highest mean MTT absorbance values across all materials, whereas exposure to the cleansing solutions generally resulted in lower values. CAD/CAM milled specimens exhibited higher MTT absorbance values under most experimental conditions, whereas conventionally heat-polymerized PMMA specimens generally exhibited the lowest values.

**Figure 3:**
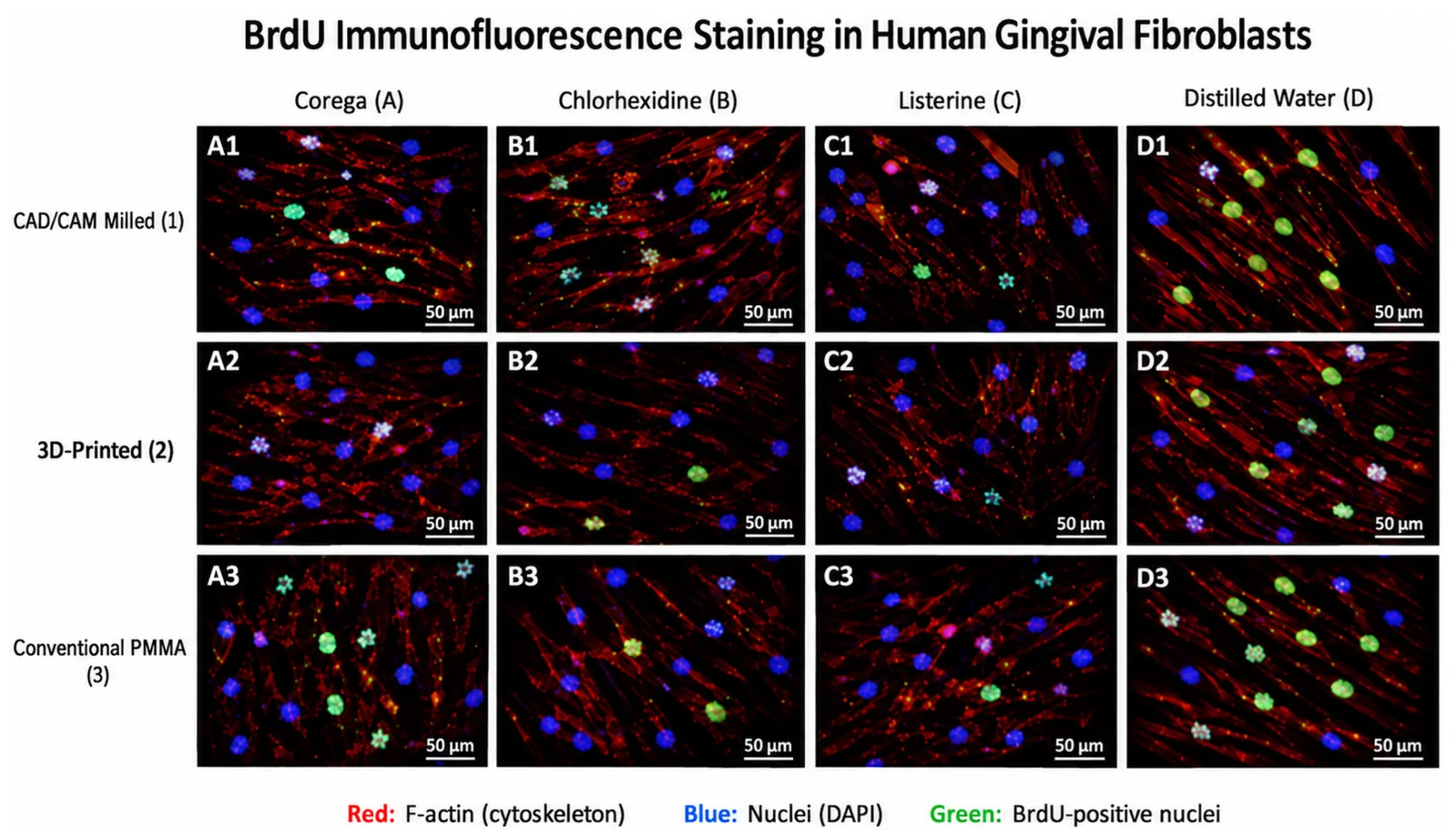
Representative composite fluorescence images of human gingival fibroblasts exposed to extracts of CAD/CAM-milled PMMA (row 1), 3D-printed methacrylate-based resin (row 2), and conventionally heat-polymerized PMMA (row 3) after immersion in Corega (**A1**–**A3**), chlorhexidine (**B1**–**B3**), Listerine (**C1**–**C3**), or distilled water (**D1**–**D3**). Images are from a separate double-staining experiment performed for qualitative illustration: BrdU-positive nuclei (green), F-actin stained with phalloidin (red), and nuclei counterstained with DAPI (blue). Representative regions of 20× images were enlarged, and the channels were merged; identical brightness and contrast adjustments were applied to all panels. Quantification of BrdU-positive nuclei was based on direct microscopic counts (Section 2.7; Figure 4), not on these images. Distilled water served as the reference cleansing condition. Scale bars: 50 µm.

**Figure 4:**
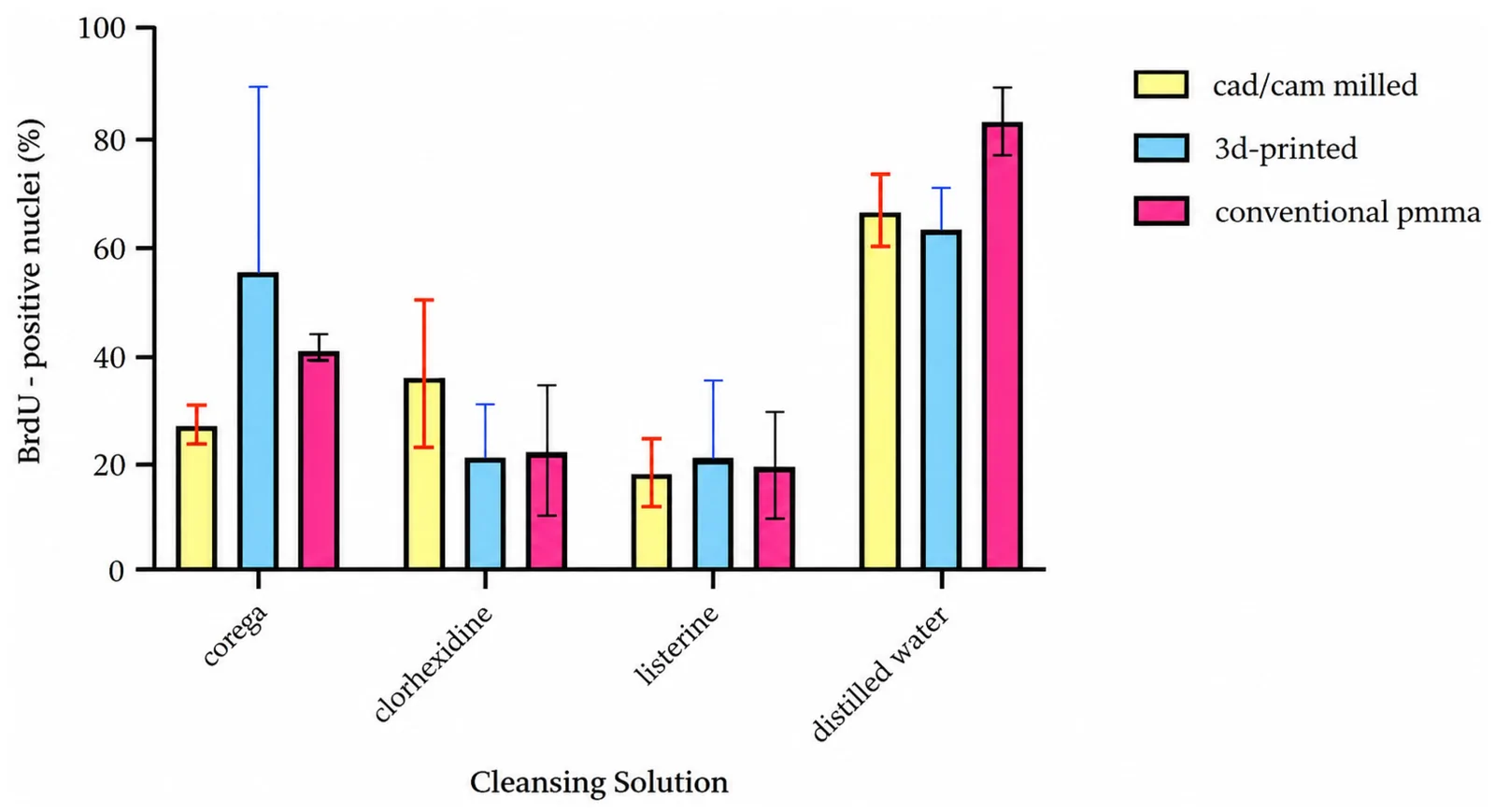
Quantitative analysis of BrdU-positive nuclei in human gingival fibroblasts exposed to extracts derived from CAD/CAM-milled PMMA, 3D-printed methacrylate-based denture base resin, and conventionally heat-polymerized PMMA following exposure of the materials to different denture cleansing solutions. The BrdU labeling index was calculated as the percentage of BrdU-positive nuclei relative to the total number of DAPI-stained nuclei. Bars represent mean values, and error bars indicate standard deviations (SD) from three independent experiments (*n* = 3). Five randomly selected microscopic fields were evaluated per experiment.

Quantitative BrdU analysis revealed a significant main effect of cleansing solution on the percentage of BrdU-positive nuclei (F(3,24) = 27.313, *p* < 0.001), whereas neither the main effect of material (F(2,24) = 0.355, *p* = 0.705) nor the material × solution interaction (F(6,24) = 2.137, *p* = 0.086) was statistically significant. The highest group mean labeling indices occurred after distilled water exposure: conventional PMMA, 82.54 ± 6.02%; CAD/CAM-milled PMMA, 66.46 ± 6.37%; and 3D-printed resin, 63.28 ± 7.27%. In Šídák-adjusted comparisons averaged across the three materials, distilled water differed from Corega (adjusted *p* = 0.0004), chlorhexidine (adjusted *p* < 0.001), and Listerine (adjusted *p* < 0.001). Corega differed from Listerine (adjusted *p* = 0.0105), but not from chlorhexidine (adjusted *p* = 0.1509); chlorhexidine and Listerine did not differ (adjusted *p* = 0.8309). BrdU ANOVA residuals departed from normality (Shapiro–Wilk *p* = 0.020). The mean-centered Levene test indicated unequal variances (*p* = 0.0018), whereas the median-centered Brown–Forsythe test did not (*p* = 0.736). Morphology and F-actin organization were evaluated qualitatively (Figure 3); BrdU labeling indices are shown in Figure 4. The two-way ANOVA results for the BrdU labeling index are summarized in Table 5. Additional representative BrdU immunofluorescence images are provided in Supplementary Figure S1.

## 4. Discussion

The present study evaluated MTT responses of human gingival fibroblasts to extracts from CAD/CAM-milled PMMA, a 3D-printed methacrylate-based denture base resin, and conventionally heat-polymerized PMMA after exposure to denture cleansing solutions. Both denture base material and cleansing solution significantly influenced MTT absorbance, and a significant interaction between these factors was observed. Quantitative BrdU immunofluorescence analysis showed a significant main effect of cleansing solution on BrdU-positive nuclei percentages, whereas the main effect of denture base material and the material × cleansing solution interaction were not statistically significant. Fibroblast morphology and F-actin organization were assessed qualitatively. As a preliminary screening study, the design allows differences among materials and cleansing conditions to be detected but not explained.

Biocompatibility is a critical consideration in the evaluation of denture base materials, as material-derived substances may influence surrounding oral tissues [24,25]. In the present study, CAD/CAM milled PMMA demonstrated the highest MTT absorbance under most experimental conditions, followed by 3D-printed specimens, whereas conventionally fabricated PMMA showed the lowest MTT absorbance. Previous studies have demonstrated favorable biological performance of digitally fabricated denture base materials, including CAD/CAM milled and 3D-printed resins [26]. Residual monomer release has also been shown to vary according to the fabrication technique and processing protocol [27,28].

Direct comparison with previous cytotoxicity studies is complicated by differences in resin composition, cell model, extraction protocol, exposure duration, MTT measurement conditions, and normalization procedures. Nevertheless, Table 6 summarizes the MTT-derived findings of the present study alongside recent studies evaluating denture base materials [20,22,29].

In comparison with previous reports, the MTT-derived viability values in the present study were generally within or above the ranges considered non-cytotoxic in the cited studies, although direct numerical comparison is limited by methodological differences. Tzeng et al. reported cell viability above 70% for all tested DLP-printed urethane-acrylate resin groups [29], whereas Krishnamoorthi et al. found no significant differences in OD or cell viability among heat-cured PMMA groups containing different proportions of recycled PMMA [22]. AlAzzam et al. reported viability values above the 70% threshold for both heat-polymerized and 3D-printed denture-base groups across the evaluated time points, although statistically significant differences were observed in certain comparisons [20]. The present findings are only partly consistent with these reports. First, Tzeng et al. [29] and Krishnamoorthi et al. [22] reported no significant differences among their tested groups, whereas significant differences among materials were detected in the present study. However, these studies primarily investigated resin formulations or modifications rather than the combined effects of manufacturing methods and cleansing solutions. Furthermore, Tzeng et al. [29] used a 24 h cell exposure, and both studies [22,29] used mouse fibroblast cell lines. AlAzzam et al. [20] evaluated both heat-polymerized and 3D-printed denture-base materials at different time points, including day 7. In their study, the unmodified 3D-printed resin showed numerically higher absorbance than the unmodified heat-polymerized PMMA at day 7, which is directionally similar to the present results. However, a statistically significant difference between these two control materials cannot be inferred from the reported values alone. Second, the mean viability of conventional PMMA after Corega (71.4%) and chlorhexidine (75.7%) exposure was close to the 70% level, with large standard deviations, so these groups should not be regarded as clearly non-cytotoxic. Third, the distilled-water groups of the milled and 3D-printed materials yielded mean values above 100% with large standard deviations; this may reflect increased metabolic activity relative to the untreated control, between-specimen variability, or the normalization procedure, and these percentages are therefore reported as descriptive values only. The 70% viability criterion of ISO 10993-5 [30] was not used to classify the materials as cytotoxic or non-cytotoxic, because the extract dilution and exposure time used here differ from the conditions specified in that standard.

CAD/CAM-milled PMMA showed higher MTT absorbance values than conventionally heat-polymerized PMMA under several tested conditions. Previous studies have demonstrated that residual monomer content and release from denture base resins may vary depending on the fabrication and polymerization conditions [28,31]. However, residual monomer content and specific leachable compounds were not quantified in the present study. Therefore, the observed differences in MTT absorbance among the tested materials cannot be specifically attributed to differences in residual monomer release based on the present findings. Likewise, the MTT findings for the 3D-printed material should be interpreted in the context of the specific methacrylate-based resin and post-polymerization protocol used in this study.

An alternative explanation that cannot be excluded is that the differences in MTT absorbance among materials reflect differences in the effective surface area available for leaching rather than intrinsic differences in material cytotoxicity. The surface-area-to-volume ratio (approximately 1.34 cm^2^/mL) was calculated from the geometric area of each disk, which does not account for surface roughness or porosity. Although all specimens were finished with the same abrasive sequence, materials produced by milling, layer-by-layer printing, and flask-based polymerization may differ in surface topography and porosity [32], which could alter extraction efficiency and therefore the amount of leachable substances reaching the cells. Because roughness, porosity, and surface morphology were not characterized, this possibility was not evaluated, and the observed differences should not be attributed to material chemistry alone.

The cleansing solution significantly influenced MTT absorbance, with higher values in the distilled water groups than in the cleanser-exposed groups. The study design does not establish which chemical or material-related factors accounted for these differences. Previous studies have assessed material properties after denture cleanser exposure. Hanno and Abdul-Monem [10] evaluated hardness, fracture toughness, water sorption, and solubility of CAD/CAM-milled and 3D-printed denture base materials. Ozyilmaz and Akin [33] investigated surface roughness, hardness, and surface topography, while Çakmak et al. [34] examined surface roughness and color changes in differently fabricated denture base materials. These outcomes were not measured in the present study and cannot be directly related to its cellular findings. Chlorhexidine and Corega generally yielded lower MTT absorbance than distilled water. Although chlorhexidine has been reported to exert cytotoxic effects on human gingival fibroblasts [35], the present extract-based design cannot determine whether any residual cleansing agent contributed to the observed responses.

The significant material × cleansing solution interaction indicates that the pattern of MTT absorbance across cleansing conditions differed according to denture base material. Because the present experiment evaluated extracts after an accelerated cumulative exposure protocol, these findings should not be interpreted as direct evidence of clinical cytotoxicity or as a basis for selecting a specific cleanser.

Quantitative BrdU analysis complemented the MTT findings by assessing DNA synthesis-associated proliferative activity. Unlike MTT absorbance, for which significant effects of denture base material and cleansing solution were observed, the BrdU labeling index showed a significant main effect of cleansing solution but not material. MTT absorbance indicates cellular metabolic activity, whereas BrdU incorporation identifies cells undergoing DNA synthesis during labeling [14,15]; accordingly, the two measures need not change proportionally. However, this explanation should be treated cautiously: in the distilled-water groups, conventional PMMA showed the lowest MTT absorbance but the highest BrdU labeling index, so the two assays did not rank the materials in the same way. Because the BrdU analysis relied on three independent experiments and showed no significant material effect, this discrepancy cannot be interpreted mechanistically. BrdU labeling indices were higher after distilled water exposure than after the tested cleansing solutions. Qualitative fluorescence imaging also suggested differences in fibroblast morphology and F-actin organization, which were not quantified.

### Limitations

This preliminary screening study has several limitations. First, its in vitro design involved only human gingival fibroblasts and cannot reproduce the full oral environment or responses of other oral cell types. Second, the accelerated exposure protocol reproduced a cumulative cleansing duration of 1825 min, but not the timing of daily clinical use. Third, TEMPO-CAD was primarily intended by its manufacturer for long-term temporary crowns and bridges, rather than denture bases. Its selection was based on previous prosthodontic reports in which Oğuz et al. fabricated denture bases from TEMPO-CAD blocks [36] and Sakarya evaluated it among CAD/CAM-milled denture base materials [37]. Therefore, the findings obtained for the milled group should not be generalized to CAD/CAM PMMA materials specifically designed for denture bases.

The results for the milled group therefore cannot be generalized to CAD/CAM PMMA disks designed specifically for denture bases. Fourth, degree of conversion, residual monomer content (including MMA), and extract composition were not measured. Thus, observed MTT and BrdU responses cannot be assigned to a particular leachable compound or polymer-conversion difference. Future work could use FTIR to assess degree of conversion, as demonstrated for denture base resins [38], and chromatographic methods to characterize residual monomers or other extract constituents [27,31]. Fifth, surface roughness, porosity, surface morphology, surface chemistry, and mechanical properties were not assessed. Because the extraction ratio was based on geometric surface area, differences in the effective surface area available for leaching among materials could not be excluded. Future investigations could combine quantitative porosity assessment [32], surface profilometry [33,34], and scanning electron microscopy (SEM) [33] to further characterize material surfaces and structural characteristics following cleansing exposure. X-ray photoelectron spectroscopy (XPS) could also be considered to investigate surface chemical composition, as previously applied to denture cleanser-exposed, titanium dioxide-coated PMMA [39]. Mechanical testing before and after cleansing could be added to evaluate material performance alongside biological assays [10]. In addition, specimens were immersed in 70% ethanol for 1 h during the initial disinfection procedure. Although this procedure was applied consistently across all experimental groups, potential ethanol-induced changes in surface characteristics and subsequent leaching behavior cannot be excluded, as these effects were not directly evaluated [21]. The extraction ratio was also lower than that recommended by ISO 10993-12 for this specimen thickness, so absolute viability values should be interpreted cautiously. Although the specimens were rinsed after cleansing exposure, the potential presence of residual cleansing agents in the subsequently prepared extracts was not chemically investigated. Future studies could incorporate appropriate chemical analyses and experimental controls to distinguish the potential contributions of material-derived leachables and residual cleansing agents to the observed cellular responses. Previous research has investigated the cytotoxicity of substances released from denture-cleanser-treated acrylic resin using an extract-based fibroblast MTT assay [12].

An additional limitation is that different extraction media were used for the two biological assays. MTT extracts were prepared using Minimum Essential Medium (MEM), whereas BrdU extracts were prepared using Dulbecco’s Modified Eagle Medium (DMEM). Differences in medium composition may influence the extraction of leachable substances and subsequent cellular responses. Therefore, differences between MTT absorbance and BrdU labeling indices cannot be attributed solely to the distinct biological endpoints assessed by these assays. Future studies should standardize the extraction medium across assays to improve comparability.

Finally, although BrdU-positive nuclei were quantified, morphology and F-actin organization were assessed qualitatively. The BrdU analysis comprised three independent experiments per group, with five microscopic fields per experiment. BrdU-positive nuclei were scored directly at the microscope, and images were not recorded for every scored field; therefore, the counts cannot be re-analyzed from archived images. In addition, the composite images in Figure 3 originate from a separate double-staining experiment and serve only as qualitative illustration. The small number of independent experiments and the departure from normality and mean-centered variance homogeneity assumptions limit the precision and robustness of parametric inferences, particularly individual pairwise comparisons. Additional independent replicates, quantitative morphometric or cytoskeletal measures, and images acquired at other magnifications could be considered in future investigations.

Future work using additional oral cell types, more clinically representative aging schedules, saliva, and biofilm models may help establish how these in vitro observations translate to denture maintenance conditions.

## 5. Conclusions

Within the limitations of this preliminary in vitro screening study, denture base material, cleansing solution, and their interaction significantly influenced MTT absorbance in human gingival fibroblasts exposed to specimen-derived extracts. CAD/CAM-milled PMMA exhibited higher overall MTT absorbance than conventional heat-polymerized PMMA, while its overall difference from 3D-printed resin was not statistically significant. Distilled water produced higher overall MTT absorbance than the tested cleansing solutions.

Quantitative BrdU immunofluorescence showed a significant overall cleansing-solution effect on BrdU-positive nucleus percentages, without statistically significant material or material × solution effects. Distilled water was associated with higher BrdU labeling indices than the tested cleansing solutions when averaged across materials. The small number of independent experiments and deviations from parametric assumptions warrant cautious interpretation. Fibroblast morphology and F-actin organization were assessed qualitatively. These in vitro observations cannot be directly extrapolated to clinical outcomes. The mechanisms underlying the observed differences were not investigated, and because the milled material (TEMPO-CAD) is not primarily intended for denture bases, the results should not be generalized to CAD/CAM denture base PMMA. Importantly, TEMPO-CAD is not primarily intended for denture base fabrication. Further studies with more independent biological replicates, chemical and surface characterization, and assessment of possible cleanser carryover are warranted.

## Figures and Tables

**Table 1:** Denture cleansing agents, manufacturers, main components, and cleansing protocols used in the study.

Group	Cleansing Agent	Manufacturer	Main Components	Cleansing Protocol
Corega	Corega Tabs	Haleon, Weybridge, UK	Sodium bicarbonate, citric acid, potassium monopersulfate, sodium carbonate peroxide, TAED, sodium lauryl sulfate	One tablet dissolved in 200 mL tap water
CHX	Klorhex 0.2%	Drogsan, Istanbul, Türkiye	0.2% chlorhexidine gluconate	30 mL, undiluted
Listerine	Listerine Cool Mint	Kenvue, Summit, NJ, USA	Essential oils (eucalyptol, thymol, menthol, methyl salicylate)	30 mL, undiluted
Control	Distilled water	—	—	Distilled water only

**Table 2:** Descriptive statistics of MTT absorbance values according to denture base material and cleansing solution.

Solution	Material	Min–Max	Mean ± SD
Corega	CAD/CAM milled	0.35–1.66	1.07 ± 0.26
	3D-printed methacrylate-based resin	0.39–1.35	0.84 ± 0.26
	Conventional PMMA	0.39–1.02	0.72 ± 0.22
Chlorhexidine	CAD/CAM milled	0.44–1.41	0.93 ± 0.24
	3D-printed methacrylate-based resin	0.38–1.35	0.81 ± 0.26
	Conventional PMMA	0.40–1.25	0.76 ± 0.27
Listerine	CAD/CAM milled	0.46–1.35	0.84 ± 0.24
	3D-printed methacrylate-based resin	0.39–1.41	0.86 ± 0.26
	Conventional PMMA	0.44–1.26	0.82 ± 0.23
Distilled water	CAD/CAM milled	0.87–1.84	1.26 ± 0.31
	3D-printed methacrylate-based resin	0.63–1.71	1.20 ± 0.32
	Conventional PMMA	0.41–1.56	0.84 ± 0.33

**Table 3:** Two-way ANOVA results for MTT absorbance values.

Source	SS	df	MS	F	*p*	Effect Size
Material	2.291	2	1.146	15.936	<0.001	0.123
Solution	2.797	3	0.932	12.971	<0.001	0.146
Material × Solution	1.295	6	0.216	3.003	0.008	0.073

df, degrees of freedom. Significant main effects were observed for denture base material and cleansing solution, as well as for the material × solution interaction.

**Table 4:** Significant Bonferroni-adjusted pairwise comparisons of MTT absorbance values.

Comparison	Mean Difference (I − J)	*p* Value
Main effect: Denture base material		
CAD/CAM milled vs. Conventional PMMA	0.238	<0.001
3D-Printed Methacrylate-Based Resin vs. Conventional PMMA	0.141	0.003
Main effect: Cleansing solution		
Corega vs. Distilled water	−0.221	<0.001
Chlorhexidine vs. Distilled water	−0.263	<0.001
Listerine vs. Distilled water	−0.256	<0.001
Interaction: Within Corega		
CAD/CAM milled vs. 3D-Printed Methacrylate-Based Resin	0.227	0.024
CAD/CAM milled vs. Conventional PMMA	0.348	<0.001
Interaction: Within distilled water		
CAD/CAM milled vs. Conventional PMMA	0.416	<0.001
3D-Printed Methacrylate-Based Resin vs. Conventional PMMA	0.353	<0.001
Interaction: Within CAD/CAM milled		
Corega vs. Listerine	0.229	0.045
Chlorhexidine vs. Distilled water	−0.326	0.001
Listerine vs. Distilled water	−0.418	<0.001
Interaction: Within 3D-Printed Methacrylate-Based Resin		
Corega vs. Distilled water	−0.353	<0.001
Chlorhexidine vs. Distilled water	−0.384	<0.001
Listerine vs. Distilled water	−0.330	0.001

Only statistically significant Bonferroni-adjusted pairwise comparisons are presented. Complete pairwise comparison results are provided in Supplementary Table S1. Mean differences are expressed as I − J. Statistical significance was set at *p* < 0.05.

**Table 5:** Two-way ANOVA results for the BrdU labeling index (%).

Source of Variation	df	F Value	*p* Value
Denture base material	2, 24	0.355	0.705
Cleansing solution	3, 24	27.313	<0.001
Material × cleansing solution	6, 24	2.137	0.086

Note: BrdU labeling indices were analyzed using two-way ANOVA with three independent experiments per material–cleansing solution combination (*n* = 3). df values are numerator and denominator degrees of freedom. Statistical significance was set at *p* < 0.05.

**Table 6:** Comparison of MTT-based cytotoxicity findings of the present study with previous studies on denture base materials.

Study	Material/Fabrication Method	Cell Type	MTT Conditions	MTT Result/Cell Viability
Present study	CAD/CAM-milled PMMA	Human gingival fibroblasts (HGF)	Specimen-derived extract; 7-day cell exposure; 570 nm	Corega: 108.3 ± 27.3%; CHX: 93.8 ± 26.0%; Listerine: 84.1 ± 25.2%; DW: 128.3 ± 33.2%
Present study	3D-printed methacrylate-based denture-base resin	HGF	Specimen-derived extract; 7-day cell exposure; 570 nm	Corega: 84.2 ± 27.7%; CHX: 81.0 ± 27.1%; Listerine: 86.7 ± 27.1%; DW: 121.7 ± 34.1%
Present study	Conventional heat-polymerized PMMA	HGF	Specimen-derived extract; 7-day cell exposure; 570 nm	Corega: 71.4 ± 23.6%; CHX: 75.7 ± 28.2%; Listerine: 82.2 ± 24.4%; DW: 84.2 ± 34.5%
Tzeng et al., 2021 [29]	DLP 3D-printed urethane-acrylate-based denture-base resins	L929 mouse fibroblasts	24 h extraction; 24 h cell exposure; MTT; 570 nm	Cell viability >70% for all tested groups; no significant difference among groups (*p* = 0.764).
Krishnamoorthi et al., 2022 [22]	Heat-cured PMMA containing 0–50 wt% recycled PMMA	L929 mouse fibroblasts	Eluate-based MTT; 570 nm	No statistically significant difference in mean OD or cell viability among tested resin groups; recycled-PMMA modification was reported as non-cytotoxic.
AlAzzam et al., 2025 [20]	Heat-polymerized PMMA (ProBase Hot), unmodified control	Human periodontal ligament fibroblasts	Extract-based MTT; 24 h, Day 3, and Day 7; 420 nm	0.79 ± 0.07 (24 h); 0.77 ± 0.02 (Day 3); 0.76 ± 0.07 (Day 7)
AlAzzam et al., 2025 [20]	3D-printed denture-base resin (NextDent Denture 3D), unmodified control	Human periodontal ligament fibroblasts	Extract-based MTT; 24 h, Day 3, and Day 7; 420 nm	0.775 ± 0.01 (24 h); 0.740 ± 0.05 (Day 3); 0.898 ± 0.02 (Day 7)

Abbreviations: CHX, chlorhexidine gluconate; DW, distilled water; HGF, human gingival fibroblasts; MTT, 3-(4,5-dimethylthiazol-2-yl)-2,5-diphenyltetrazolium bromide; OD, optical density; PMMA, polymethyl methacrylate. Note: Cell viability values for the present study are presented as mean ± SD (*n* = 20 per material–cleansing solution combination). Viability percentages were calculated using common reference values obtained from the mean absorbance of 20 untreated control wells and 20 cell-free blank wells across plates. Values from previous studies are presented as reported in the original publications and were not recalculated. Because the studies differed in material composition, cell type, extraction protocol, exposure duration, MTT measurement conditions, and normalization procedures, the findings are intended for descriptive comparison and should not be interpreted as directly quantitatively equivalent.

## Data Availability

The original contributions presented in this study are included in the article/Supplementary Materials. Further inquiries can be directed to the corresponding author.

## Supplementary Materials

The following supporting information can be downloaded at: https://www.mdpi.com/article/10.3390/ma19214761/s1, Figure S1: Representative BrdU single-staining (Texas Red) images of human gingival fibroblasts exposed to resin-derived extracts; Table S1: Complete Bonferroni-adjusted pairwise comparisons of MTT absorbance values according to denture base material and cleansing solution; Table S2: Raw MTT absorbance data for the 12 material–cleansing solution groups.
