# Supplementary material for: Cytotoxic Effects of Milled, 3D-Printed, and Conventional Methacrylate-Based Resins After Exposure to Denture Cleansers: A Preliminary In Vitro Screening Study
Source: Materials (Basel). 2026 Nov 9;19(21):4761. doi: 10.3390/ma19214761 (PMC13764704; doi:10.3390/ma19214761)
Supplement: Supplementary file 1 [file materials-19-04761-s001.zip › Figure_S1.pdf]

Supplementary Figure S1

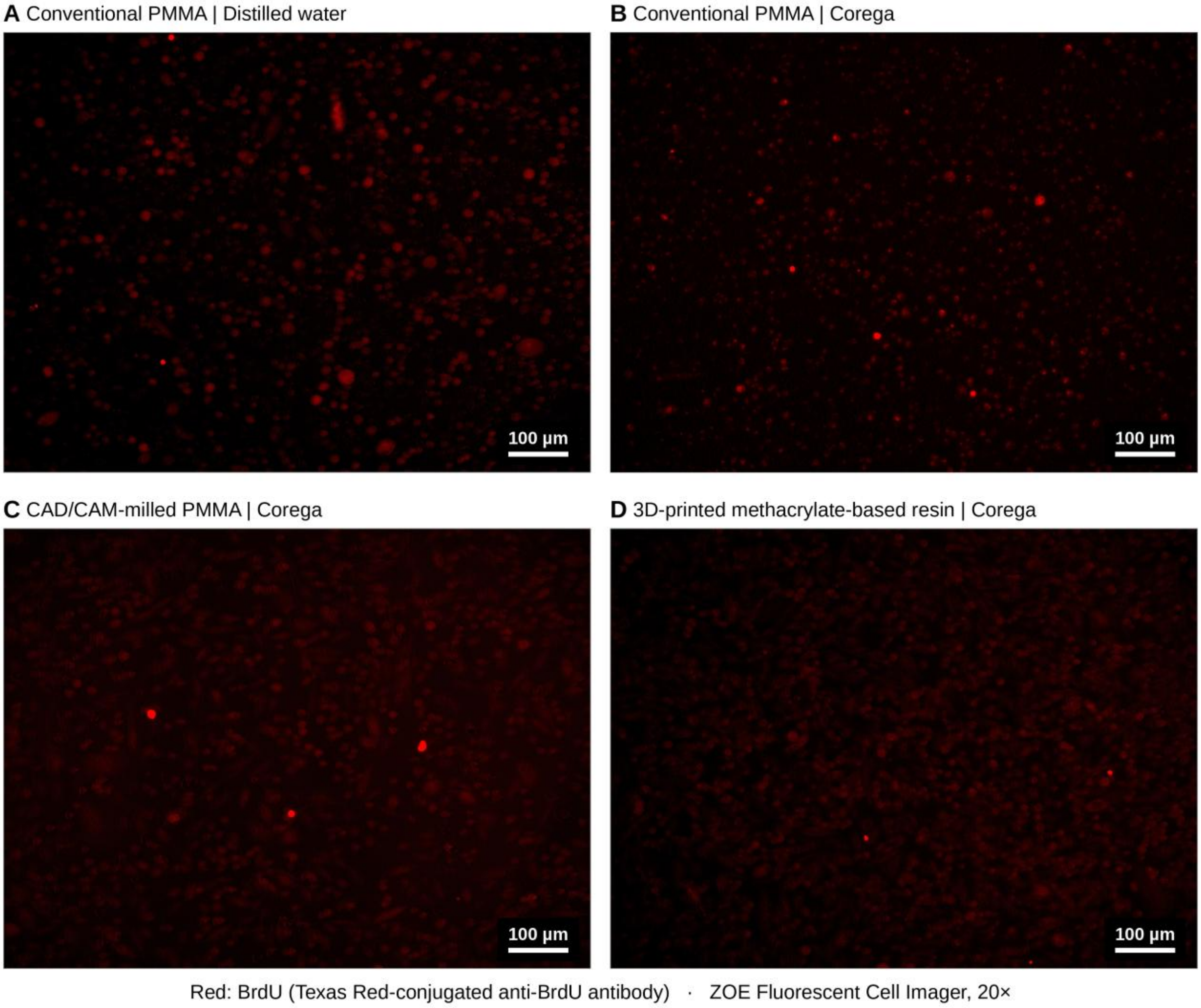

Figure S1. Representative BrdU single-staining images of human gingival fibroblasts from the BrdU quantification experiments, acquired with a ZOE Fluorescent Cell Imager (Bio-Rad) at 20×. BrdU was detected with a Texas Red-conjugated anti-BrdU antibody (red). (A) Conventional PMMA, distilled water; (B) conventional PMMA, Corega; (C) CAD/CAM-milled PMMA, Corega; (D) 3D-printed methacrylate-based resin, Corega. No brightness or contrast adjustments were applied; panels were resized to identical dimensions, and scale bars were redrawn at the original calibrated length. Scale bars: 100 µm.
