# Supplementary material for: Cytotoxic Effects of Milled, 3D-Printed, and Conventional Methacrylate-Based Resins After Exposure to Denture Cleansers: A Preliminary In Vitro Screening Study
Source: Materials (Basel). 2026 Nov 9;19(21):4761. doi: 10.3390/ma19214761 (PMC13764704; doi:10.3390/ma19214761)
Supplement: Supplementary file 1 [file materials-19-04761-s001.zip › Table_S1_Bonferroni.pdf]

Supplementary Table S1. Complete Bonferroni-adjusted pairwise comparisons of MTT absorbance values according to denture base material and cleansing solution.

Group	I	J	Mean Difference (I–J)	P
**Overall material comparisons**	Milled	3D print	0.097	.071
	Milled	Lunacast	0.238	<.001*
	3D print	Lunacast	0.141	.003*
**Overall cleansing solution comparisons**	Corega	Chlorhexidine	0.042	1.000
	Corega	Listerine	0.035	1.000
	Corega	Distilled water	−0.221	<.001*
	Chlorhexidine	Listerine	−0.008	1.000
	Chlorhexidine	Distilled water	−0.263	<.001*
	Listerine	Distilled water	−0.256	<.001*
**Milled**	Corega	Chlorhexidine	0.137	.642
	Corega	Listerine	0.229	.045*
	Corega	Distilled water	−0.189	.162
	Chlorhexidine	Listerine	0.092	1.000
	Chlorhexidine	Distilled water	−0.326	.001*
	Listerine	Distilled water	−0.418	<.001*
**3D print**	Corega	Chlorhexidine	0.031	1.000
	Corega	Listerine	−0.023	1.000
	Corega	Distilled water	−0.353	<.001*
	Chlorhexidine	Listerine	−0.054	1.000
	Chlorhexidine	Distilled water	−0.384	<.001*
	Listerine	Distilled water	−0.330	.001*
**Lunacast**	Corega	Chlorhexidine	−0.041	1.000
	Corega	Listerine	−0.102	1.000
	Corega	Distilled water	−0.121	.935
	Chlorhexidine	Listerine	−0.061	1.000
	Chlorhexidine	Distilled water	−0.080	1.000
	Listerine	Distilled water	−0.019	1.000
**Corega**	Milled	3D print	0.227	.024*
	Milled	Lunacast	0.348	<.001*
	3D print	Lunacast	0.121	.465
**Chlorhexidine**	Milled	3D print	0.121	.467
	Milled	Lunacast	0.170	.138
	3D print	Lunacast	0.049	1.000
**Listerine**	Milled	3D print	−0.024	1.000
	Milled	Lunacast	0.017	1.000
	3D print	Lunacast	0.042	1.000
**Distilled water**	Milled	3D print	0.063	1.000
	Milled	Lunacast	0.416	<.001*
	3D print	Lunacast	0.353	<.001*

Mean differences (I–J) and Bonferroni-adjusted P values are presented. Statistical significance was set at $P < .05$. *Statistically significant.
